# Search for Nutritional Fitness Traits in a Biological Pest Control Agent *Harmonia axyridis* Using Comparative Transcriptomics

**DOI:** 10.3389/fphys.2019.01148

**Published:** 2019-09-18

**Authors:** Tingting Zhang, Yulong He, Jianyong Zeng, Lisheng Zhang, Fanrong Zeng, Jianjun Mao, Guocai Zhang

**Affiliations:** ^1^School of Forestry, Northeast Forestry University, Harbin, China; ^2^Business School, Huaqiao University, Quanzhou, China; ^3^Key Laboratory for Biology of Plant Diseases and Insect Pests, Ministry of Agriculture, Institute of Plant Protection, Chinese Academy of Agricultural Sciences, Beijing, China

**Keywords:** artificial diets, ingredients, transcriptome analysis, reproduction, nutritional, development

## Abstract

*Harmonia axyridis* is an important natural predator used in the biological control of insect pests. Vitellogenin (Vg) supplementation to artificial diet can improve fecundity of *H. axyridis*, however, the effects of Vg on physiology of *H. axyridis* at the molecular level is unclear. This study investigated the effects of Vg on the physiology (digestive enzyme activities) and transcriptome patterns by feeding *H. axyridis* adults with treatment (artificial diet with Vg supplement) and control (artificial diet supplemented with bovine serum albumin (BSA). The transcriptome sequencing yielded 43.94 Gb of clean data, and 3,946 differentially expressed genes (DEGs) – including 93 upregulated and 3,853 downregulated genes between the treatment and control. Six DEGs related to development and digestive enzyme were chosen for quantitative real-time PCR (qRT-PCR) to validate the accuracy of the RNA-seq results and confirmed that the transcriptome analysis yielded reliable results. The Vg supplement has increased activities of digestive enzymes and related genes expression in *H. axyridis*. The transcript level of digestive enzyme genes (apolipoprotein D and phosphoenolpyruvate carboxykinase) were much higher in adults fed on diet supplemented with Vg compared with that of the control.

## Introduction

*Harmonia axyridis* (Pallas) is an omnivorous predator that was widely introduced in North America and Europe as early as 1916 (Gordon, 1985; Adriaens et al., 2003). This polyphagous predator feeds on aphids, coccids (Mcclure, 1987; Hodek and Honěk, 2009), lepidopteran eggs (Ferran et al., 1984) and other insects (Koch et al., 2003). Due to its large predation, good adaptability and strong fecundity, this insect is widely used for biological control purposes (De Bach and Rosen, 1991; Ferran et al., 1996; Yasuda et al., 2000). To reduce the use of and adverse effects of chemical pesticides, insect pest control by natural enemy insects has received widespread attention. Mass rearing is important for the commercial production of beneficial insects used in the biological control of insect pests and has become a promising industry (Lenteren et al., 1997; Greany and Carpenter, 1998). However, a natural prey diet is unable to sustain a population of *H. axyridis* over an entire year because the environmental conditions might limit the supply of prey. It is thus necessary to implement an artificial diet to rear this beneficial insect as supplemental predators for the effective field control of pests. Coudron et al. (2002) indicated that the cost of rearing *Podisus maculiventris* on an artificial larval diet is similar as that associated with the use of natural prey. Zhang et al. (2015) studied an artificial diet that could be used as larval food in the mass rearing of *H. axyridis*. Similarly, other researchers also reported three artificial diets used to rear *H. axyridis* (Castro-Guedes et al., 2016).

The digestive enzymes are important in insects for food digestion and nutrient absorption. In insects, the food is ingested through the food canal into the alimentary canal, where it is further digested and absorbed by enzymes such as trehalase and lipase. Trehalase is very important for the use of sugar, energy production and macromolecular biosynthesis in insects (Friedman, 1978). Also, lipase plays an important role in the lipid metabolism and absorption, and related to the growth and development of insects (Chapman, 1998; Dottorini et al., 2007). The nutrients digested by enzymes play a crucial role in the development and reproduction of the insects. Tufail and Takeda (2008) reported that Vg is major nutrition source for the embryo development. Many researchers have showed that Vg is related with growth and development of occytes and egg production (Zeng et al., 1997). The amount and quality of food directly influence various biological aspects of Coccinellidae (Dixon, 2000), and so a lack of nutrients could affect their growth and development (Hodek et al., 2012). In fact, food permits the complete development and reproduction of Coccinellids. The nutrients in an artificial diet also impact the degree of ovarian maturation and the regulatory mechanisms for oogenesis (Adams, 2000). One goal in mass insect rearing is to use cost-effective artificial diets. Cheng et al. (2017) found that the artificial diet for adults was superior to any previously developed formulas and might thus have potential for improving the artificial diet used for the mass rearing of *Chrysopa septempunctata*. These studies reveal that nutrient addition is beneficial to the metabolism and optimization of the artificial diet and thus affects the physiology and reproduction of *H. axyridis*. However, most current artificial diets for *H. axyridis* result in low fecundity, and the methods used to evaluate their diet are limited in terms of their biological category, thus, the other alternative method is necessary to test the effects of the diet formulation on insect performance, such as diet optimization by measuring a few preselected biochemical and physiological parameters. In addition, the techniques of comparative transcriptomics may be used to help effectively evaluate the artificial diets for mass rearing of insects. Based on a study showing that nutrition affects gene expression patterns (Yocum et al., 2006), the study of these patterns not only allows the exploration of the insects’ response to changes in their food stream but also offers information on diet limitations. Zou et al. (2013) compared the transcriptome of *Arrma chinensis* fed an artificial diet with that of *Antheraea pernyi* pupae and found that many metabolic pathways related to nutrition were upregulated in the diet-fed *A. chinensis*. Some diet-regulating genes play an important role in reproduction and longevity as found by Alaux et al. (2011) in that nutrition influences genes related to longevity and the production of some antimicrobial peptides. Chen and Hsiao (1984) found that a delay in oviposition or an increased developmental rate might decrease the intrinsic rate. Liu et al. (2015) cloned the complete Vg cDNA sequence from a Neuropteran species, *C. septempunctata* Wesmael, and studied the vitellogenn functions, they found that RNAi mediated by injection of dsRNA depleted *CsVg* transcripts, significantly reduced egg production and decreased egg hatching rate. Du and Zeng (2016) analyzed time-series RNA-seq data from the *H. axyridis* adult ovary to identify development-related genes. Zhang T. et al. (2017) further explored the effects of Vg fragment on development and reproduction *H. axyridis*, they found that the Vg fragment significantly increased the egg production of *H. axyridis*. The influence of artificial diet supplemented with Vg on physiology and the transcriptome of *H. axyridis* may be used to further understand the action of nutrients on *H. axyridis* at molecular level and improve artificial diet for this insect. Therefore, we have studied the effects of Vg supplement in an artificial diet on *H. axyridis* using comparative transcriptome. We hypothesized that the addition of Vg supplement could increase *H. axyridis* enzyme related gene expression levels, and then increase enzyme activities and improve nutritional digestion and absorption and therefore increase reproduction of *H. axyridis*. The main goals of this study were to understand the influence of Vg supplement on *H. axyridis* physiology (enzyme activities) and egg reproduction, assess the molecular-level effects of Vg on *H. axyridis* using RNA-seq technology. The study will obtain the abdomen transcriptomes of *H. axyridis* adults fed diet supplemented with Vg (treatment) or BSA (control) on the fifth day after oviposition and performed an analysis of generate accurate data, and identify nutritional and development-related genes. The information obtained from the transcriptome-wide study of mRNA in the *H. axyridis* abdomen may be used to improve the artificial diet for the growth and reproduction of *H. axyridis*.

## Results

### Transcriptome Sequencing and Read Assembly

The transcriptome sequencing of the *H. axyridis* abdomen yielded 43.94 Gb of clean data. Libraries of controls samples (C1 and C2) and the libraries treatments samples (T01, T02, and T03) were collected (Only 5 samples, two BSA controls (C1 and C2) and three Vg treatments (T1, T2, and T3), were analyzed by DEGs (Anders and Huber, 2010), C3, one of control samples was degraded and not included for analysis). Five libraries containing clean reads (C1: 20,632,283; C2: 29,972,500; T01: 31,985,871; T02: 35,389,508; T03: 30,517,849) were generated for analysis after cleaning and checking. The percentages of GC contents were analyzed in five libraries (C1: 39.88%; C2: 40.13%; T01: 39.45%; T02: 39.81%; T03: 39.53%). The five libraries of percentage ≥ Q30 were 87.54, 91.43, 90.68, 91.37, and 91.43%, respectively (Table 1). After assembly, the total numbers of 107,939 transcripts and 75,717 unigenes were obtained, and the N50s of the transcripts and unigenes were 1,922 and 1,400 bp, respectively. Means length of the transcripts and unigenes were 1009.31 and 767.48 bp (Table 2). The raw transcriptome reads were deposited in the NCBI SRA database (accession number: SRP104023).

**TABLE 1:** Statistics of the sequencing data.

**Samples**	**Clean reads**	**Clean data**	**GC Content (%)**	**%=Q30**
C1	20,632,283	6,095,457,898	39.88%	87.54%
C2	29,972,500	8,908,858,910	40.13%	91.43%
T01	31,985,871	9,446,577,644	39.45%	90.68%
T02	35,389,508	10,479,909,588	39.81%	91.37%
T03	30,517,849	9,012,385,962	39.53%	91.43%

*Samples, name of the sample; clean reads, total number of paired-end reads in the clean data; GC content, percentage of G and C bases among the total bases; and % ≥ Q30, percentage of bases with quality value ≥ 30.*

**TABLE 2:** Sequencing data statistics.

**Length range**	**Transcript**	**Unigene**
200-300	32,454 (30.07%)	29,864 (39.44%)
300-500	21,231 (19.67%)	16,784 (22.17%)
500-1000	21,932 (20.32%)	14,055 (18.56%)
1000-2000	17,264 (15.99%)	8,644 (11.42%)
2000 +	15,058 (13.95%)	6,370 (8.41%)
Total Number	107,939	75,717
Total Length	108,944,081	58,111,525
N50 Length	1,922	1,400
Mean Length	1009.31	767.48

### Annotation of Unigenes

For functional annotation, we received 27,992 unigenes annotated using Blastx tool against NCBI Integrated Nr databases (Nr), 14,025 unigenes were annotated in the GO annotation databases, 14,803 unigenes were annotated in the KEGG annotation databases, 23,524 unigenes were annotated in the Pfam annotation database, 10,535 unigenes were annotated in the COG, and 22.803 unigenes were annotated in the KOG annotation databases, respectively. A total of 36,668 unigenes were annotated based on the selection of parameters with an *E*-value ≤ 1e-10 (Table 3).

**TABLE 3:** Statistics of the unigene annotations.

**#Anno_Database**	**Annotated_ Number**	**300=length < 1000**	**Length = 1000**
COG_Annotation	10535	4001	4655
GO_Annotation	14025	5320	6430
KEGG_Annotation	14803	5918	6325
KOG_Annotation	22803	8884	9388
Pfam_Annotation	23524	9275	10690
Swissprot_Annotation	28316	11364	12289
eggNOG_Annotation	33884	13860	12085
Nr_Annotation	27992	11158	12266
All_Annotated	36668	15183	12738

### Analysis of Differentially Expressed Genes (DEGs)

**FIGURE 1:**
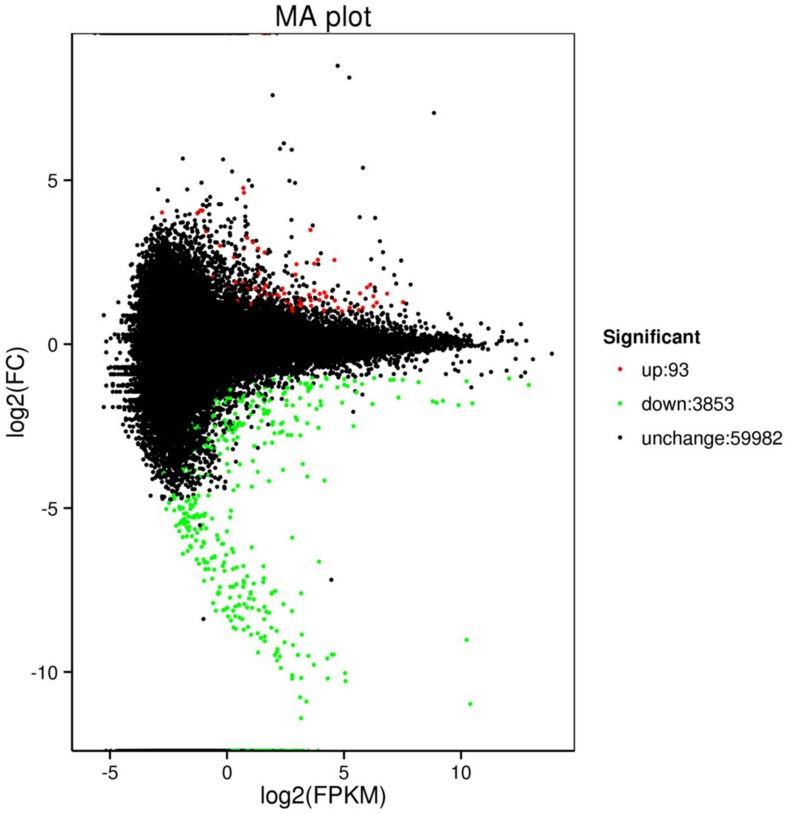
Scatter diagrams representing the comparisons of the genome-wide expression profiles of control and treatment. Note: Each green point represents a decrease in gene expression, each red point represents an increase in gene expression, and each black point represents no significant difference in gene expression.

The comparison of control (C2 and C2) with treatment (T01, T02, and T03) identified 3,946 DEGs, including 93 upregulated genes and 3,853 downregulated genes (Table 4). The MA plot clearly shows the overall distribution of gene expression abundance and the various differences between the two sets of sample groups. Each black point represents a non-significant difference in gene expression, each red point indicates an upregulate expressed gene, and each green point indicates a downregulate expressed gene. Abscissa: log2 (FPKM) represents the logarithm of the mean value of the expressions in the two sample groups. Ordinate: log2 (FC) represents the logarithm of the multiple of gene expression difference between the two sample groups (Figure 1). All of the obtained DEGs were combined into one DEG database (Supplementary Dataset File S1).

**TABLE 4:** Number of differentially expressed genes.

**DEG_Set**	**All_DEG**	**Upregulated**	**Downregulated**
C1_C2_vs_T01_T02_T03	3946	93	3853

*The threshold value for significance in the comparison of the control (C1 and C2) with the treatment (T01, T02, and T03) was FPKM fold change > 2 and FDR < 0.05.*

### Functional Annotation and Enrichment Analysis of DEGs

**FIGURE 2:**
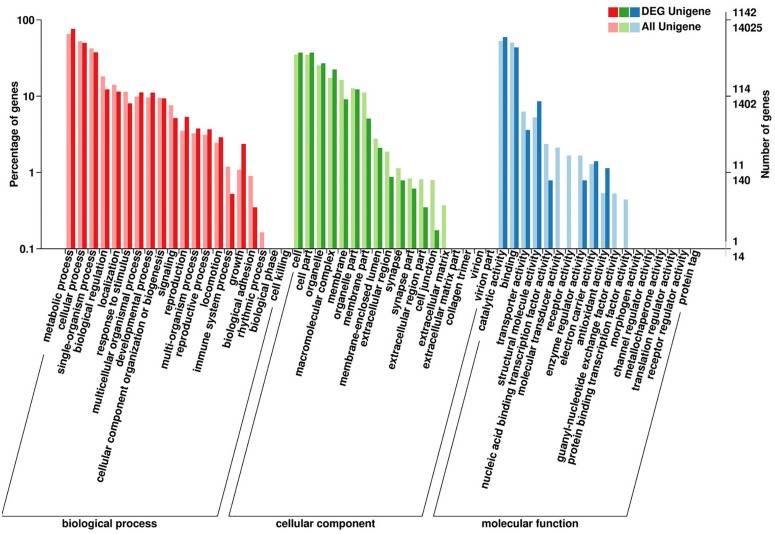
Summary of the annotations of differentially expressed genes in *H. axyridis*. The GO classifications according to involvement in biological processes, cellular components and molecular functions are shown.

**FIGURE 3:**
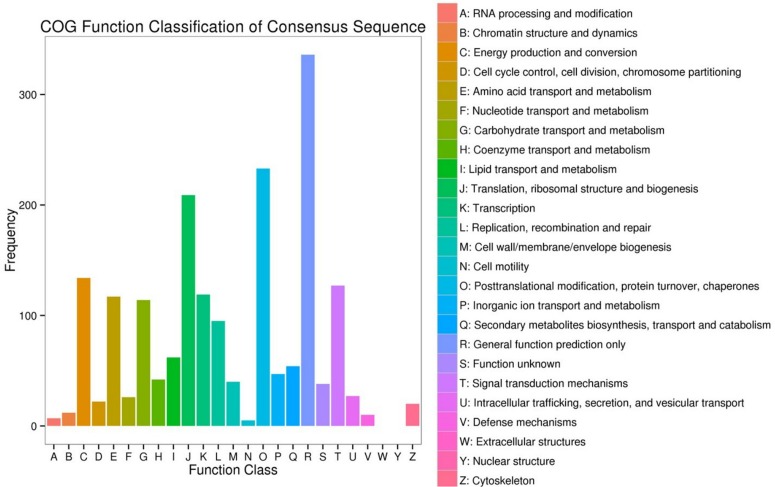
Clusters of Orthologous Groups (COG) functional classification. The 1,471 sequences were classified into 25 COG categories.

The DEGs enrichment was classified into 56 different groups belonging to three main categories of GO annotation: they are biological process (22901 unigenes), cellular component (6700 unigenes) and molecular function (8896 unigenes) (Figure 2 and Supplementary Dataset Files S2–S4). Within the biological process, the three most common categories were metabolic process, cellular process and single-organism process. The COG database can be used for the direct classification of gene products, and 1,471 sequences had a COG classification. Among the 25 COG categories, the cluster for “General function prediction only” constituted the largest group (336, 22.84%), followed by “Posttranslational modification, protein turnover, chaperones” (233, 15.84%), “Translation, ribosomal structure and biogenesis” (209, 14.21%), “Energy production and conversion” (134, 9.11%) and “Signal transduction mechanisms” (127, 8.63%). In contrast, “Extracellular structures” (0, 0%), “Nuclear structure” (0, 0%), “Cell motility” (5, 0.34%) and “RNA processing and modification” (7, 0.48%) represented the smallest groups (Figure 3).

### Quantitative Real-Time PCR Validation of the DEGs Results

**FIGURE 4:**
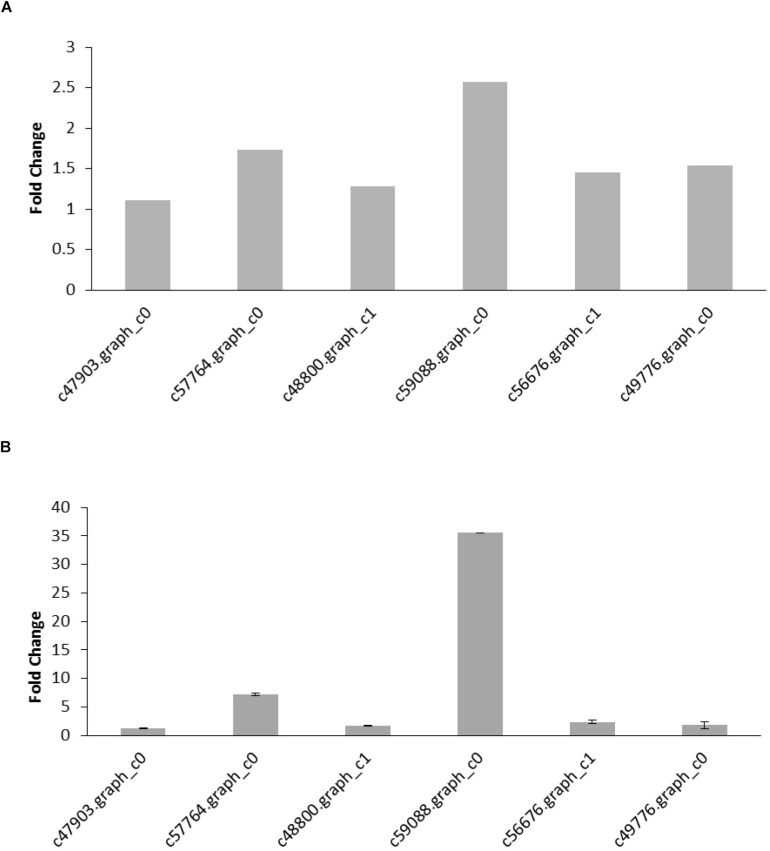
**(A)** DEGs identified from the transcriptome analysis. The fold change of the gene was calculated as a log_2_ value shown on y-axis. **(B)** qRT-PCR results of gene expression. The values are expressed as the means ± SEs of three independent biological replicates.

To validate the DEGs determined from the transcriptome results, six reproduction and nutrition-related genes were selected from the results for qRT-PCR analysis. The six pairs of primers were designed to validate the RNA-seq data (Table 5). β-actin was used as an internal control for normalization. The thresholds were significantly differences in six genes expression (FDR < 0.05; FPKM > 2). The qRT-PCR results of six genes were confirmed the expression pattern with a trend of upregulation. Hexamerin 3 precursor and apolipophorins showed the upregulated significantly with the up-trend, and the other genes were slightly upregulated in the raise expression in the qRT-PCR results. The expression levels of six target genes were consistent with the qRT-PCR and RNA-seq data (Figure 4). Thus, qRT-PCR analysis confirmed that the transcriptome analysis identified the correct change direction and yielded reliable results.

**TABLE 5:** Sequences of the primers used for the qRT-PCR analysis of six randomly selected genes.

**Gene ID**	**Homologous function in Nr**	**Primer (5′-3′)**	**Product size**	**Expression level**	**Accession no.**
β-actin		CTATGTCGGAGCCATCACT	112		
		AGCAGTTGTAGCTTCTCCGT			
c47903.graph_c0	pleiotrophin isoform X1	ACGAAGACCATCCAGAAG	129	up	MH341578
		TTCACAAGAGGCATCACT			
c57764.graph_c0	hexamerin 3 precursor	CCAAGTAGACGCCAAGTA	132	up	MH341579
		CCGCCATATACACCAGAT			
c48800.graph_c1	collagen alpha-1(IV) chain	GGCGTTCCTGGTATTATTG	189	up	MH341580
		GGTGTAGTCTTCCTGTTCT			
c59088.graph_c0	apolipophorins	CAATGCGAAGACAGAAGTT	158	up	MH341581
		CGAAGTGAATATCTACAGTATGG			
c49776.graph_c0	apolipoprotein D	CGACCTCAGTTAATATGTTAGTAA	190	up	MK886791
		GATGTAGATTGTTGCTTCAGA			
c56676.graph_c1	phosphoenolpyruvate carboxykinase	TAACTCAAGGTAAGAACAC	158	up	MK886792
		AAAGTATCATCAAATAACATTAGA			

### Effects of Vg Supplement on Physiology and Related Genes of *H. axyridis*

**FIGURE 5:**
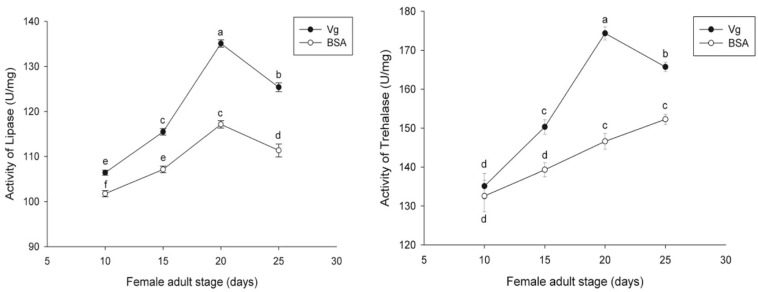
*H. axyridis* lipase and trehalase activity during the female adult stage after treatment with the Vg- and BSA-supplemented artificial diets. The significance of the differences in the enzyme activities were analyzed by SPSS. The enzyme activities as shown as the means ± SEs and were found to be significantly different (*P* < 0.0001).

**FIGURE 6:**
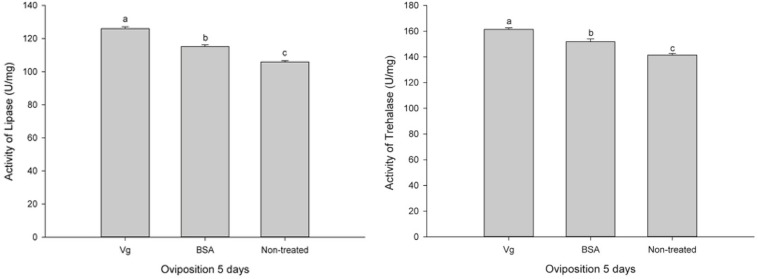
*H. axyridis* lipase and trehalase activities on the fifth day after female oviposition after treatment with the Vg- and BSA-supplemented artificial diets. The significance of the differences in the enzyme activities were analyzed by SPSS. The enzyme activities as shown as the means ± SEs and were found to be significantly different (*P* < 0.0001).

**FIGURE 7:**
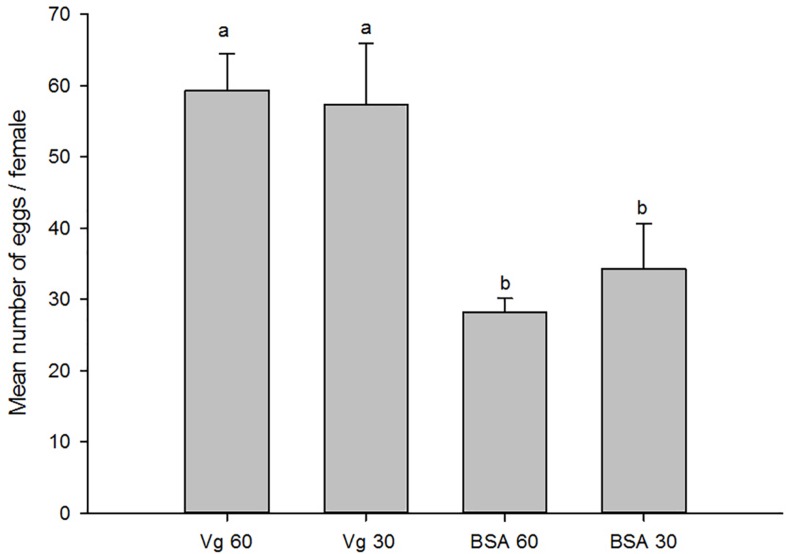
Total *H. axyridis* egg production in groups of the Vg- and BSA-supplemented artificial diets during 1 month period. The data were analyzed by SPSS. The egg production was shown as the means ± SEs.

The effects of the Vg supplement on physiology (enzyme activities and reproduction) of *H. axyridis* were investigated. The lipase and trehalase activities during the female adult stage and on the fifth day after female oviposition after fed with the Vg (treatment) and BSA (control)-supplemented artificial diets were determined, respectively. The activities of the treatment groups were markedly different from those obtained from the control group for the lipase (*F* = 641.666; *df* = 16, 23; *p* < 0.0001) and trehalase (*F* = 155.867; *df* = 16, 23; *p* < 0.0001) (Figure 5) during the female adult stage. Similarly, the activities of lipase (*F* = 99.219; *df* = 2, 8; *p* < 0.0001) and trehalase (*F* = 40.227; *df* = 2, 8, *p* < 0.0001) in the treatment groups were significant differences on the fifth day after oviposition compared with the control group (Figure 6). The results from this study indicated that Vg supplement had effect on *H. axyridis* reproduction. The data showed that the total eggs in the groups treated with 60 μg/mL of Vg fragment were significantly higher than those groups treated with 60 μg/mL of BSA (*F* = 6.991; *df* = 3, 23; *P* = 0.002) (Figure 7) during the 1 month period. The total eggs in the groups treated with 30 μg/mL of Vg fragment were also much higher (1.67 times) than those in the groups treated with 30 μg/mL of BSA (Figure 7).

The effects of the Vg supplement on physiology related gene transcription levels of *H. axyridis* were also determined. The 3 growth and development-related and 2 enzyme-related genes were identified using the unigenes annotation databases.

The gene id, homologous function in Nr, full length, product size, *E*-value and accession number are summarized (Table 6). According to the nr annotations, these genes belong to the following groups: two genes belong to heat shock protein gene group, one gene is in serine protein group, another gene is related to hydrolase group, one gene belongs to transport and catabolism gene group.

**TABLE 6:** Development and enzyme-related differentially expressed genes were shown in nr.

**Gene ID**	**Homologous function in Nr**	**Full length**	**Product size**	***E*-value**	**Accession no.**
c52950. graph_c0	Serine protease 33	Yes	1812	2.28E-10	MN241440
c49623. graph_c0	heat shock protein 70 B2-like	Yes	1970	3.45E-14	MN241441
c52099. graph_c0	heat shock protein 90	Yes	926	4.32E-17	MN241442
c47043. graph_c1	Abhydrolase domain-containing protein 4-like isoform X1	Yes	2197	9.35E-05	MN241443
c53557. graph_c1	ATP-binding cassette sub-family A member 3-like (Tribolium castaneum)	Yes	805	0.009619	MN241444

## Discussion

The nutritional composition of food is a key factor that influences the development and reproduction of insect species (Bonte et al., 2010). An assessment of predator insect survival and reproductive performance indicates whether an artificial diet has potential for use in the mass rearing of the insect (Xie et al., 2017). Previous studies showed that adult reproductive performance was unfavorably affected by restricted food supply during the larval stages (Xie et al., 2015a, b). Also, Niijima et al. (1977) developed an artificial diet for *H. axyridis*, they found that an artificial diet does not assist in egg production; however, it does sustain adult *H. axyridis.* In the present study, we used different supplement samples mixed with a successful artificial diet (Zhang S. et al., 2017) to compare the differences between the Vg and BSA proteins effects through a transcriptome analysis, the gene expression profiles between insects fed the Vg and BSA were compared by Illumina sequencing to obtain insights into the enzyme activities, nutritional absorption, energy metabolism and reproduction of *H. axyridis*. The Illumina sequencing of five samples yielded 43.94 Gb of clean data, and the data were assembled into 75,717 unigenes. Functional annotation results were obtained for 36,668 of the assembled unigenes. The gene structures were analyzed using a unigene library, and 4,637 SSR markers were obtained from an SSR analysis. Based on the RNA-seq results, approximately 62 DEGs in the *H. axyridis* abdomen were related to reproductive development. A previous study suggested that the Vg fragment can increase the reproduction of *H. axyridis* (Zhang T. et al., 2017). These 62 DEGs could provide new insights for our understanding of the nutrigenomics of *H. axyridis* affected by components of artificial diet such as Vg supplement.

Furthermore, the results from this study revealed that different insect artificial diet components showed the influence on gene regulation. We selected six DEGs related to nutrition and development, which found different gene expression levels due to different component in artificial diet. The mRNA levels of these genes were notably higher in insects fed the artificial diet with Vg-supplement than in those fed the diet with BSA. The six genes are pleiotrophin isoform X1, collagen alpha-1(IV) chain, hexamerin 3 precursor, apolipophorins, Phosphoenolpyruvate carboxykinase and Apolipoprotein D. The c47903.graph_c0 was annotated as pleiotrophin isoform X1 and might have participated in the development; previous studies showed that pleiotrophin is involved in the migratory processes of neurons during brain development (Nobuaki and Masaharu, 1998). The c48800.graph_c1 was annotated as collagen alpha-1(IV) chain and is similar to collagen alpha-1 (X), which is a major avian eggshell membrane (ESM) structural constituent (Cordeiro and Hincke, 2016). The c57764.graph_c0 was annotated as a hexamerin 3 precursor; the hexamerins of insects belong to a growing protein superfamily that also includes the arthropod hemocyanins and prophenoloxidases and the hexamerin receptors discovered in Diptera (Beintema et al., 1994; Burmester and Scheller, 1996; Telfer and Kunkel, 2003). The hexamerins of hexapods are derived from hemocyanins with whom they share a similar protein structure, whereas insect hexamerins serve as storage proteins (Terwilliger et al., 1999; Burmester, 2002). Levenbook and Bauer (1984) investigated hexamerin function during metamorphosis, and Burmester (1999) recognized the inter-order hexamerin relationships and the assignment of distinct hexamerin classes conserved within single insect orders. The c59088.graph_c0 was annotated as apolipophorins and participates in lipid transport vehicles; the fat body serves as the site of apolipophorin I (apoLp-I), apolipophorin II (apoLp-II) and lipid synthesis and well as the site of lipid storage and lipoprotein assembly. Depending on the insect species, lipophorins are released as high-density lipoproteins into the haemolymph (Prasad et al., 1986; Venkatesh et al., 1987; Capurro and Bianchi, 1990; Weers et al., 1992; Heusden et al., 1998). Phosphoenolpyruvate carboxykinase (PEPCK,c56676.graph_c1) has been indicated to catalyze a rate-limiting step in lactate gluconeogenesis in hepatocytes from fasted rats (Rognstad, 1979). Our results revealed that the expression of PEPCK, an enzyme belonging to the lyase family that participates in the gluconeogenesis metabolic pathway, was upregulated. Specifically, PEPCK converts oxaloacetate into phosphoenolpyruvate and carbon dioxide (Chakravarty et al., 2005; Méndez-Lucas et al., 2013). Gluconeogenesis is used by humans and many other animals to maintain blood glucose levels and avoid low levels of glucose (hypoglycemia), and it is used for glycogen degradation (glycogenolysis). PEPCK may indirectly enhance glucose levels and provide nutrients to adult insects. Apolipoprotein D (ApoD, c49776.graph_c0) is belongs to the lipocalin family (Sánchez et al., 2002),which is a secreted glycoprotein with many putative functions including lipid transport. The Vg protein may stimulate nutritional-related gene expression, followed by more protease activities and the utilization of lipids and proteins in *H. axyridis*. This study showed that an artificial diet supplemented with Vg protein could be more effective for rearing *H. axyridis*.

Similar to all oviparous animals, insects supply their eggs with proteins, carbohydrates, and other resources to provide sustenance for the developing embryo. Trehalase serves as an energy reserve in insects, and changes in nutrition, physiological and environmental conditions lead to different concentrations of trehalase in the insect haemolymph. If insect development and life activities require energy, the stored trehalase can be converted into glucose (Elbein et al., 2003). Zeng and Cohen (2001) found that certain foods can induce enzyme activity, and other researchers have reported that food quality affects insect biochemistry (Shapiro and Legaspi, 2006). Vg is a major protein in insect eggs (Tufail and Takeda, 2008), and the promotion of Vg synthesis indicates an increase in protein requirements. The data from this study showed that upregulate gene was related to enzyme activities, and artificial diets with different components might stimulate related gene expression, which increases enzyme activities. In insects, food is digested and absorbed by enzymes such as trypsin (Zeng and Cohen, 2000; Zeng et al., 2002). The results from this study support our hypothesis and the presence of Vg supplement in artificial diets alter the enzyme activity of *H. axyridis*, and then increase the digestion of nutrients in the artificial diet, and subsequently increase the reproduction of *H. axyridis*.

## Materials and Methods

### Insect Rearing and Treatment

Throughout all of its developmental stages, the first colony of *H. axyridis*, was collected from the suburbs of Beijing and reared in a growth chamber (RXZ, Ningbo, China) with a climate-controlled incubator maintained at 25 ± 1°C with a 16-h light: 8-h dark photoperiod and 70 ± 5% relative humidity. The artificial diet to feed *H. axyridis* included major components of pork liver, eggs, sugar, yeast and so on (the artificial diet contains protein, sugar and lipid, which constitute approximately 10.9, 1.95, and 1.72% of the feed, respectively (Zhang T. et al., 2017). The full-length *H. axyridis* V*g* cDNA was cloned by RT-PCR. A Willerbrand factor type D (VWD) domain were expressed and purified by the clone Vg gene was characterized. Vg fragment contains specific Vg conserved domain (VWD) and can express specific protein related amino-acides. The insects belonging to the treatment groups were fed with Vg supplement (developed and expressed by this research), and the control groups were fed with BSA supplement (purchased from JiangChen, Beijing, China). Abdomen samples were collected 5 days after oviposition. The six samples of control with three replications (C1, C2, and C3) and the treatment with three replications (T01, T02, and T03) were collected and send out for analysis by BioMarker (China, Beijing). The Illumina Solexa deep-sequencing technology was used for the transcriptome sequencing of the *H. axyridis* abdomen samples.

### cDNA Library Construction and Illumina Sequencing for Transcriptome Analysis

Five days after oviposition, the total RNA from *H. axyridis* adult females was extracted using the Tranzol reagent according to the manufacturer’s instructions. The control and treatment group samples were collected from the first abdominal ganglia to the tail end. Genomic DNA was digested with DNase I (Transgene, Beijing, China), and the RNA integrity was determined by agarose gel electrophoresis, which revealed clear bands for 18S and 28S. For ligation to sequence adapters, the cDNA was purified and repaired, and A bases were added to the 3′-ends.

### Unigene Annotation

Basic Local Alignment Search Tool (BLAST) (Altschul et al., 1997) software was used to compare the unigenes to the Integrated Nr database (Deng et al., 2006), Swiss-Prot (Apweiler et al., 2004), Gene Ontology (GO) (Ashburner et al., 2000), Clusters of Orthologous Groups (COG) (Tatusov et al., 2000), eukaryotic Orthologous Groups (KOG) (Koonin et al., 2004), eggNOG4.5 (Huerta-Cepas et al., 2016), Kyoto Encyclopedia of Genes, and Genomes (KEGG) (Kanehisa et al., 2004) were used to annotate the unigenes using KOBAS2.0 (Xie et al., 2011). The amino acid sequences of the unigenes were predicted, and annotation information for the unigenes was then obtained through comparison with the Protein family (Pfam) (Finn et al., 2014) database using HMMER software (Eddy, 1998). Gene function annotation was performed using the following databases: Nr^1^, COG^2^, Pfam^3^, KOG^4^, Swiss-Prot^5^, KEGG (see text footnote 5), and GO^6^.

### DGE Analysis

The DESeq (Anders and Huber, 2010) was used to analyse the differential expression between the control and treatment groups in this study to obtain the expression gene sets from above groups. During the differential expression analysis, the Benjamini-Hochberg method was adopted to correct the *p*-value obtained by the original hypothesis test, namely, the FDR (False Discovery Rate), which was used as the key indicator for screening the differentially expressed genes. In the screening process, fragments per kilobase of exon model per million mapped reads (FPKM) fold change > 2 and a false discovery rate (FDR) < 0.05 were used as the thresholds to identify significant differences in gene expression.

### Real-Time Quantitative RT-PCR

The primers for the target and reference genes were designed using the online primer3 program (version 4.0.0)^7^ and are listed in Table 5. The total RNA from female adults was isolated and treated with RNase-free DNase I at 37°C for 30 min using the DNase I kit (Takara, Dalian, China). A reaction volume of 20 μl, which contained 200 nM of each forward and reverse primer, cDNA produced from 2 μg of total RNA, 8 μl of nuclease-free water, and 10 μl of 2X iTaq universal SYBR Green Supermix (BIO-RAD, CA, United States) was used. The real-time PCR analysis of 200 ng of total RNA was performed using a 7500 Real-Time PCR System (Applied Biosystems, Carlsbad, CA, United States). The qPCR reaction conditions were as follows: 95°C for 2 min followed by 40 cycles of 95°C for 15 s and 60°C for 1 min. The levels of the target genes were compared with those of the housekeeping gene β-actin using the 2^–ΔΔCt^ qPCR method. The means and standard errors for each time point were obtained from the average of four independent samples. The individual animals were randomized into two treatment groups, and three biological replications of each treatment were performed.

### Effects of Vg Supplement on Physiology and Related Genes of *H. axyridis*

The effects of the Vg supplement on physiology (enzyme activities and reproduction) of *H. axyridis* were investigated. The lipase and trehalase activities and the related gene transcription levels of *H. axyridis* were also determined. The trehalase activity was measured using a trehalase ELISA kit (SU-B97117, Collodi, Quanzhou, China). Five days after spawning, the Vg protein- and BSA protein-fed female adults were weighed and individually grounded in liquid nitrogen. Every 100 mg of insect tissue was suspended in 1 ml of sample diluent. All standards and samples were added in duplicate to a microtiter plate. The wash buffer (20X) was diluted with 19 volumes of deionized or distilled water, and 50 μl of the sample was added to the appropriate wells. The blank wells did not contain any standard or sample. One hundred microliters of enzyme conjugate was added to the standard and sample wells (not to the blank wells). The plate was then covered with adhesive strips, incubated at 37°C for 60 min and washed four times, and 50 μl each of substrate A and B were added to each well. The plate was then gently mixed and incubated at 37°C for 15 min. If the color in the wells was green or not uniform, the plate was gently tapped to ensure thorough mixing. Fifty microliters of stop solution was then added each well, and the color changed from blue to yellow. Within 15 min, the optical density (OD) was read at 450 nm using a microtiter plate reader (Flexstation 3, CA, United States). The levels of lipase and trehalase activity were detected using the Insect Lipase (Trehalase) ELISA kit (SU-B97122, Collodi, Quanzhou, China), according to the procedures described for the measurement of activity. The effects of the Vg on *H. axyridis* egg production determined as follows: the Vg supplement in different concentrations (30 and 60 μg/mL) were added into the artificial diet to feed *H. axyridis* adults in the treatment groups. The control groups were supplemented with equivalent concentrations of BSA proteins (Guangda, Beijing, China). The total eggs in the treatment and control groups within 1-month period by each pair of adults were recorded. The experimental design was total randomized with six replications.

### Statistical Analysis

The differential gene expression between the control and treatment groups in this study was analyzed by DESeq (Anders and Huber, 2010). The qRT-PCR results, enzyme activity measurements and egg production data were analyzed by SPSS. The data are shown as the means ± SEs of three independent biological replicates.

## Author Contributions

JM and FZ designed the study. TZ and YH performed the experiments. JZ and LZ analyzed the data. TZ, GZ, and FZ wrote the main text of the manuscript and prepared all the figures. FZ and TZ revised the manuscript. All authors reviewed the manuscript.

## Conflict of Interest Statement

The authors declare that the research was conducted in the absence of any commercial or financial relationships that could be construed as a potential conflict of interest. The handling Editor declared a shared affiliation, though no other collaboration, with several of the authors TZ, JM, FZ, and LZ at the time of review.

## Footnotes

1 https://ftp.ncbi.nih.gov/blast/db/

2 http://www.ncbi.nlm.nih.gov/COG/

3 http://pfam.xfam.org/

4 https://ftp.ncbi.nih.gov/pub/COG/KOG

5 http://www.genome.jp/kegg/

6 http://www.geneontology.org/

7 http://primer3.ut.ee/
